# P-501. Concerns and Opinions of People Currently or Previously on Oral and Long-Acting Injectable Forms of HIV Preexposure Prophylaxis (PrEP)

**DOI:** 10.1093/ofid/ofae631.700

**Published:** 2025-01-29

**Authors:** Alice Schaack, Maxwell Allamong, Frances Hung, Richard Barfield, Mehri McKellar

**Affiliations:** Duke University, Durham, North Carolina; Duke Institute for Survey Methodology, Durham, North Carolina; Duke University, Durham, North Carolina; Duke University, Durham, North Carolina; Duke University Hospital, DURHAM, North Carolina

## Abstract

**Background:**

Long acting injectable (LAI) preexposure prophylaxis (PrEP) is an effective tool that has the potential to increase PrEP uptake and adherence. However, the majority of studies investigating interest and opinions surrounding LAI PrEP were theoretical, done prior to its approval. It is important to better understand peoples’ opinions about LAI PrEP as uptake increases.

**Methods:**

We administered a cross-sectional electronic survey to clients at an urban PrEP clinic in the US South. Eligible participants were 18+ years and had previously taken or been prescribed oral or LAI forms of PrEP. Participants were recruited at clinic appointments or through a link sent via the electronic medical record. Participants were asked about their preferences and concerns about oral and LAI PrEP and barriers to access PrEP.

**Results:**

Between January 10-April 5, 2024, 110 participants completed the survey: 73 during in-person appointments and 37 via online links. Of those, 22 were currently using LAI PrEP, 71 oral PrEP, and 17 were not currently on a form of PrEP. Participants were mostly men (89.1%), and mean age was 38 (range 17-78). 54.5% of respondents were White, 30% Black, and 14.5% Latinx. Most participants preferred an injection versus a daily pill (100% of people already on LAI, 46.5% of people on oral PrEP, and 58.8% of people not currently on PrEP). The most common concerns about PrEP for all groups were side effects, cost of medication, and cost of clinic visits and tests. Of people taking oral PrEP, 59.2% had not switched to injectable either because it was not offered to them, or they were not aware of it. The most common reasons for stopping PrEP amongst people not currently using either form were side effects and no longer needing PrEP.

**Conclusion:**

LAI PrEP is desired by many, but not all, people who are currently or have previously taken PrEP. Common concerns about PrEP include side effects and cost of medication and/or clinic visits. People on oral PrEP are frequently not offered LAI or do not know it exists. These data demonstrate the importance of improving LAI PrEP accessibility and offering people choice between PrEP methods.

**Disclosures:**

**Mehri McKellar, MD**, Gilead: Grant/Research Support

